# Fast, quantitative, murine cardiac ^19^F MRI/MRS of PFCE-labeled progenitor stem cells and macrophages at 9.4T

**DOI:** 10.1371/journal.pone.0190558

**Published:** 2018-01-11

**Authors:** Christakis Constantinides, Mahon Maguire, Eileen McNeill, Ricardo Carnicer, Edyta Swider, Mangala Srinivas, Carolyn A. Carr, Jurgen E. Schneider

**Affiliations:** 1 Departments of Cardiovascular Medicine, Radcliffe Department of Medicine, University of Oxford, Oxford, United Kingdom; 2 Department of Tumor Immunology, Radboud University Medical Center, Radboud University, Nijmegen, The Netherlands; 3 Department of Physiology, Anatomy, and Genetics, University of Oxford, Oxford, United Kingdom; Universitatsklinikum Wurzburg, GERMANY

## Abstract

**Purpose:**

To a) achieve cardiac ^19^F-Magnetic Resonance Imaging (MRI) of perfluoro-crown-ether (PFCE) labeled cardiac progenitor stem cells (CPCs) and bone-derived bone marrow macrophages, b) determine label concentration and cellular load limits, and c) achieve spectroscopic and image-based quantification.

**Methods:**

Theoretical simulations and experimental comparisons of spoiled-gradient echo (SPGR), rapid acquisition with relaxation enhancement (RARE), and steady state at free precession (SSFP) pulse sequences, and phantom validations, were conducted using ^19^F MRI/Magnetic Resonance Spectroscopy (MRS) at 9.4 T. Successful cell labeling was confirmed using flow cytometry and confocal microscopy. For CPC and macrophage concentration quantification, in vitro and post-mortem cardiac validations were pursued with the use of the transfection agent FuGENE. Feasibility of fast imaging is demonstrated in murine cardiac acquisitions in vivo, and in post-mortem murine skeletal and cardiac applications.

**Results:**

SPGR/SSFP proved favorable imaging sequences yielding good signal-to-noise ratio values. Confocal microscopy confirmed heterogeneity of cellular label uptake in CPCs. ^19^F MRI indicated lack of additional benefits upon label concentrations above 7.5–10 mg/ml/million cells. The minimum detectable CPC load was ~500k (~10k/voxel) in two-dimensional (2D) acquisitions (3–5 min) using the butterfly coil. Additionally, absolute ^19^F based concentration and intensity estimates (trifluoroacetic-acid solutions, macrophages, and labeled CPCs in vitro and post-CPC injections in the post-mortem state) scaled linearly with fluorine concentrations. Fast, quantitative cardiac ^19^F-MRI was demonstrated with SPGR/SSFP and MRS acquisitions spanning 3–5 min, using a butterfly coil.

**Conclusion:**

The developed methodologies achieved in vivo cardiac ^19^F of exogenously injected labeled CPCs for the first time, accelerating imaging to a total acquisition of a few minutes, providing evidence for their potential for possible translational work.

## Introduction

Implantation of stem cells (SCs) has provided a methodological pathway that promises cardiac tissue regeneration and structural and functional improvements following injury. The basic approach of SC therapy involves the direct transplantation of cells, followed by their migration, differentiation, and proliferation, ultimately attaining homing and engraftment. However, while the feasibility of SC technologies has been proven, efficacy is still in question [1].

Within the realm of SC therapies, non-invasive imaging and tracking of labeled SCs, and their functional impact, has taken a prominent role in recent years. The visualization of the implanted SCs to define optimal therapy strategies (dose, timing, delivery) using pre-labeled cells with fluorescent probes [2], transduced expression of fluorescent proteins [3], or iron oxide particles (MPIOs) [4], and their assessment for temporal label persistence, has become a subject of intense research. Over the past decade, nanoparticles (NPs) containing perfluoro-crown-ethers (PFCE) have led to direct tracking and quantification of exogenously labeled cell populations [5, 6, 7, 8] with ^19^F magnetic resonance imaging (MRI).

Despite the implementation of ^19^F MRI early on in the development of MRI, exploitation efforts had languished until recent years [5, 6, 9, 10]. The resurgence of interest in ^19^F imaging arose further to initiatives in molecular imaging, and capitalized on the exogenously injected fluorine’s 100% abundance, and the high relative sensitivity and contrast with respect to the ^1^H nucleus. The lack of endogenous fluorine provides fluorinated labels an added advantage as tracking agents. Consequently, the technique has found applicability in cellular labeling and tracking applications in vivo [5, 11], with potential for translational value [12].

Furthermore, prior applications were confined to either direct injections of neural SCs [13], immune cells [6, 7, 9], hematopoietic SCs [14], or on direct intravascular administrations of NP emulsions [15, 16, 17, 18, 19, 11]. Correspondingly, there have been no prior reported ^19^F MRI preclinical applications in normal or infarcted hearts using exogenously administered, labeled progenitor SCs, while direct applications of other types of SCs in the rodent heart have been limited [14].

Prior efforts attempted to optimize fluorine acquisitions in terms of speed, evoked MR signal, and cellular detectability [11], by focusing on spectroscopy [17, 18, 19] or on dedicated pulse sequences [20–26], and by selecting imaging parameters that elicited maximum signal responses, despite the prohibitively long acquisition times. To our knowledge, there is no prior ^19^F MRI study on the use of labeled cardiac progenitor cells (CPCs) (previously used to show efficacy of regeneration and cardiac functional improvements [27]). Certainly, lacking are also detailed relaxometric studies in these cells post-labeling.

We present a comprehensive methodology that applies ^19^F MRI aiming to achieve: a) fast imaging of PFCE-labeled CPCs within clinically relevant times (of the order of a few minutes) in the in vivo mouse, b) determination of detection limits of label cellular load with clinically applicable surface and volume coils, and c) spectroscopy and image-based quantification validated in phantoms, CPCs, labeled bone-marrow-derived murine macrophages, and in the post-mortem mouse. The stated objectives were investigated based on theoretical and simulation comparisons of pulse sequence performances, in vitro relaxation value characterization of PFCE-labeled CPCs, experimental concentration validations, and post-mortem and in vivo applicability of the imaging approach in the cardiac and skeletal muscles of the C57BL/6 mouse.

## Materials and methods

### Animal ethics

All procedures were in accordance with the Home Office (UK) guidelines under The Animals (Scientific Procedures) Act, 1986 (Permit Number: PIL30/3322), the European Animal Research Directive, and with local institutional guidelines. All surgery and live animal imaging was performed under isoflurane (ISO) anesthesia, and all efforts were made to minimize suffering. Animals were euthanized using cervical dislocation.

### Cardiac progenitor stem cells and bone marrow-derived macrophages

#### Nanoparticle synthesis

Particles were synthesized in accordance to previously published methodologies [7]. All particles were then extensively washed with distilled water and lyophilized for 2–3 d. For the cell labeling, nanoparticles were synthesized with the addition of fluorescent dye (Atto647, ATTO-TEC, GmbH, Germany) to the organic phase. Prior ^19^F Magnetic Resonance Spectroscopy (MRS) characterization of PFCE NP labels confirmed the presence of a single spectral peak at -91.8 ppm (with respect to CFCl_3_) [11].

#### Cell isolation

Cardiac progenitor cells (CPCs, comprising either cardiosphere-derived (CDC) or collagenase-trypsin (CT)) were isolated from adult, C57BL/6, green fluorescent protein (GFP) positive or GFP negative, mouse atria, using standard protocols [28], and were maintained in Iscove’s Modified Dulbecco’s Medium (IMDM) media (Thermo Fisher Scientific, UK). CDCs have been previously used to show efficacy of regeneration and cardiac functional improvements [27], while CTs have been recently shown to express similar cardiac phenotypic characteristics to CDCs [29]. Additionally, bone marrow-derived macrophages were cultured from bone marrow harvested from C57BL/6 mouse hindlimbs. Bone marrow cells were washed with phosphate buffer solution (PBS, Sigma-Aldrich, UK), passed through a cell strainer to produce a single cell suspension, and differentiated for a week in petri-dishes in Dulbecco’s modified eagle’s medium (DMEM) that contained L-cell conditioned media. At that point, adherent bone-marrow derived macrophages were harvested. They were subsequently washed with PBS and were re-suspended in pellets in improved minimal essential medium (OPTIMEM).

#### Cellular culture and labeling

Cells were plated in IMDM solutions and incubated with PLGA-PFCE-Atto647-containing NPs for approximately 24 h before isolation and pelleting. Addition of a fluorescent dye (Atto647) allowed independent flow cytometry and confocal microscopy validation studies. Cell pellet suspensions (CPCs or macrophages) were maintained in media (IMDM or OPTIMEM) and were subsequently used for MRI, flow cytometry, or confocal microscopy, after fixation in 2% methanol-free paraformaldehyde solution (Thermo Scientific Pierce, UK) mixed with PBS (1:7 v/v).

#### FuGENE labeling

The commercially available DNA transfection agent FuGENE (Promega, Madison, WI, USA) was used to label the CPCs (both CT and CDC cells in separate cultures) with the NPs (using 25 μl of FuGENE in ~10^6^ cells). FuGENE was pre-mixed and incubated with the NPs before cell transfection for ~20 min. Cells were then labeled overnight [30].

#### Confirmation of cellular label uptake and viability

Successful labeling was confirmed with a CyAn ADP flow cytometer (Beckman Coulter, USA) using control and labeled cell samples. Cellular viability was determined with the Trypan Blue exclusion assay, directly after labeling, and at the completion of MRI studies (wherever applicable), using a cell counter.

#### High-content epifluorescence imaging

Live cells were plated in 6-well plates and stained with Calcein (CellTrace^™^ Calcein Red-Orange, ThermoFisher Scientific, UK) and Hoechst (ThermoFisher Scientific, UK) for cytoplasmic and nuclear high-content imaging (Operetta, Perkin-Elmer, UK). Fluorescence was assessed based on Atto647. The Operetta’s Harmony software was used for image analyses. Imaging was based on a randomized field analysis methodology that covered each of the studied wells.

#### MRI/MRS

All experiments were conducted on a 9.4 T Agilent scanner equipped with a DirectDrive console and a 1000 mT/m actively shielded gradient set (internal diameter = 60 mm) (Agilent Technologies, USA). For comparative tests (pulse sequences, radiofrequency (RF) coils), the same acquisition parameters and total acquisition times were used.

#### Aqueous and cellular phantoms

Twenty-four cylindrical (15–50 ml) phantoms (1–100 mM), containing trifluoro-acetic acid (TFA), PFCE NPs, and labeled CPCs or macrophages, were used to test RF coil responses at 9.4 T (without, and with the use of adiabatic excitations), determine detection limits (TFA, NP solutions mixed in water and IMDM, labeled cells), and for image- and spectroscopy-based quantification.

#### RF coils

Coils comprising an eight-rung, low-pass, quadrature birdcage (diameter = 34 mm), an 40×20 mm^2^ butterfly constructed on a 28 mm diameter former, and a 5 (diameter) ×8 (length) mm^2^ solenoid prototypes, were constructed. All coils were tuned at 375.88 MHz (fluorine resonance), and were matched to 50 Ω. The broad frequency response of the coils (3dB range spanning a few tens of kHz) permitted imaging on both the ^1^H and ^19^F nuclei.

Spoiled gradient echo (SPGR) imaging allowed surface coil comparisons (without, and with the use of adiabatic excitation, as described below), using aqueous TFA phantoms (range of concentrations = 0.5–100 mM) with the following imaging parameters: TR = 6.3 ms, TE = 3.2 ms, flip angles = 50° (non-adiabatic), and 190° and 205° (adiabatic) (with comparable power settings for non-adiabatic and adiabatic acquisitions), NEX = 8, BW = 6 kHz, field-of-view (FOV) = 40×40 mm^2^, slice thicknesses (ST) = 2 mm, matrix = 32×32. Mid-axial profiles were obtained from reconstructed images and compared.

The butterfly coil was used for murine studies owing to its increased B_1_ detection sensitivity. The increased B_1_ homogeneity of the birdcage and solenoid coils allowed the a) assessment of the minimum detectable number of ^19^F atoms (and the fluorine content of the PLGA-NPs), and b) the estimation of relaxation times.

#### Adiabatic excitation

Phantom studies (six Eppendorf vials containing aqueous TFA solutions at 5–10 mM) were conducted without (Gaussian RF pulse excitation) and with the use of hyperbolic adiabatic full-passage (HS-AFP) RF pulses [31] using the butterfly coils to determine ultimate concentration detection limits and RF B_1_ penetration. Since HS-AFP pulses are non-ideal in achieving proper slice selection, three-dimensional (3D) adiabatic acquisitions were performed, whereby excitation of a thick tissue slab was accompanied by imaging of only a relatively smaller tissue area (relevant to the injected labeled CPCs).

Adiabatic pulse parameters were chosen appropriately to achieve adiabaticity, yet maintaining power to comparable levels as those used for Gaussian (non-adiabatic) excitations [31] (^1^H MRI: TR/TE = 13.6/1.72 ms, flip angle = 50° (versus 180° for HS-AFP adiabatic excitation), NEX = 16, BW = 50 kHz, FOV = 40×40 mm^2^, ST = 3 mm, matrix = 128×128; ^19^F MRI: TR/TE = 8.7/4.4 ms, flip angle = 50° or 190/205° for HS-AFP, NEX = 8 or 1248, BW = 4 or 8 kHz, FOV = 40×40 mm^2^, ST = 2 mm, matrix = 32×32, BW HS-AFP = 1.35 kHz, resolution = 0.4 μs, cutoff = 2 or 4%).

#### Simulations

SPGR, rapid acquisition with relaxation enhancement (RARE), and fid/echo steady state free precession (fid-SSFP, echo-SSFP) sequences were simulated in accordance with steady-state, closed-form, signal and signal-to-noise ratio (SNR) equation formulations, as described in the S1 Appendix. Parametric SNR maps were generated in MATLAB (Version 2010b, Mathworks, Natick, MA, USA) using typical ^19^F relaxation times and imaging parameters for TFA solutions and CPCs, as these were determined in this study. Estimated SNR values for SPGR and SSFP sequences were normalized to the maximum signal over the entire parametric space. SNR normalization in the case of RARE adhered to the recent analysis presented by Mastropietro et al. [25].

#### Pulse sequence comparison

Extensive prior in vivo preclinical work has favored RARE imaging [25], and more recently bSSFP [24]. The choice of the optimal pulse sequence for ^19^F MRI was based on experimental work using the homogeneous birdcage coil by comparing the SNR responses of SPGR, RARE, and SSFP sequences, based on 2D acquisitions of a 100 mM TFA solution. The imaging parameters were: (SPGR/SSFP: TR/TE = 6.7 and 6.8/3.4 ms, flip angle = 50/30°, NEX = 988/580, 32×32, FOV = 40×40 mm^2^, ST = 2 mm, BW = 6 kHz, and RARE: TR/TE = 1100/10.5 ms. NEX = 96, NEX = 1024, 32×32, ETL = 32, FOV = 40×40 mm^2^, ST = 2 mm, BW = 6 kHz, total acquisition time = 3.5 min).

SNR maps were generated by dividing the reconstructed images by the noise SD, estimated from background regions using standard methodologies. Given the relatively large SNR values (>10) of reconstructed images for cell and phantom studies, no bias corrections for the magnitude reconstruction were applied.

#### Relaxation measurements

To allow SNR optimization and direct image-based quantification, T_1_ and T_2_ measurements of aqueous TFA, NaF, NP solutions, and labeled (without and with the use of FuGENE) CPCs, were conducted with the birdcage/solenoid coils using conventional inversion recovery and Carr-Purcell-Meiboom-Gill (CPMG) pulse sequences, with the following imaging parameters: a) T_1_: TR = 5–8 s, 512 points, NEX = 2–16, BW = 4 kHz; b) T_2_: TR = 5 s, TE = 2 ms, 2048 points, NEX = 8, BW = 4 kHz.

For the relaxation measurements of CTs, cells were suspended in Eppendorf tubes and were maintained in IMDM media in ice-cold baths at 4°C and under normoxic conditions (normal oxygen tension). The tubes were then allowed to reach room temperature before measurements were conducted. The partial pressures of oxygen (pO_2_) of the tested solutions were not monitored during experiments.

In comparison to the T_1_ values, the T_2_ values of labeled CT cells were not quantified owing to the low ^19^F signal elicited from these labeled cells, often leading to exceedingly long acquisition times using nonlocalized spectroscopy that may lead to compromised oxygenation status and viability.

#### ^19^F detection threshold in TFA and nanoparticle solutions

To assess the MRI detection threshold and the optimal labeling dose (SPGR vs. SSFP), a a) TFA, and b) a phantom with PFCE label solutions were imaged with the birdcage coil using conventional ^1^H SPGR (TR/TE = 4.9/2.47ms, flip angles = 20°, NEX = 1, BW = 50 kHz, FOV = 40×40 mm^2^, ST = 2 mm, matrix = 128×128) and ^19^F SPGR and SSFP sequences (TR/TE = 4.9–6.37/2.47–3.2ms, flip angles = 50–60°, NEX = 512, BW = 4–6 kHz, FOV = 40×40 or 60×60 mm^2^, ST = 2 mm, matrix = 32×32). Both phantoms were constructed with 0.7 and 1.7 ml Eppendorf tubes containing: a) one 100 mM TFA reference standard (middle vial) and five TFA solutions (1–100 mM), b) a 25 mM TFA reference standard (middle vial) and six PLGA-PFCE solutions at different NP concentrations (2.5–10 mg/ml, or equivalently, 0.32–3.2 mM).

#### Detection threshold in labeled cells in vitro

Multiple CT cells (1, 0.75, 0.5, 0.25 million cells) were seeded, and labeled with FuGENE with NPs at a concentration of 10 mg/ml/million cells. The cells were subsequently suspended, maintained in IMDM media, and placed in multiple Eppendorf tubes for imaging using SPGR with the butterfly coil (^1^H: TR/TE = 62.34 ms, flip angle = 50°, NEX = 2, BW = 20 kHz, FOV = 40×40 mm^2^, slice thickness = 2 mm, 8 slices, matrix = 128×128; ^19^F: TR/TE = 8.54/4.29ms, flip angle = 180°, NEX = 1024, BW = 6 kHz, FOV = 40×40 mm^2^, slice thickness = 20 mm, matrix = 32×32, pulse width (HS-AFP) = 3 ms, BW = 1.35 kHz, resolution = 4 μs, and 4% cutoff. For MRS, the solenoid acquisitions used the following parameters: TR = 500 ms, 512 points, NEX = 256, BW = 20 kHz.

#### MRS and image-based concentration quantification

Implementation was achieved in phantom TFA solutions, in labeled CPCs, and in the post-mortem mouse following intracardial administration of CPCs using a setup that could be used in vivo.

In vitro quantification: Direct MRS and SPGR-image-based quantification was implemented using the birdcage coil and the multivial TFA phantom described above, based on region-of-interest (ROI) estimation. A reference calibration curve was generated with four TFA solutions (25–100 mM), whereby signals were estimated in a blinded fashion and referenced against a standard of known concentration (middle vial) (^19^F: TR/TE = 6.21/1.75, flip angle = 20°, NEX = 256, BW = 8 kHz, FOV = 40×40 mm^2^, ST = 5 mm, axial, matrix = 32×32).

Labeled CPCs: This effort was extended in CPCs (cell densities of 0.5, 0.75, 1, and 2 million) and in macrophages (cell densities ~2–6 million) using the solenoid (both cell types) and butterfly (CPCs using adiabatic excitation) coils. Imaging and quantification was achieved with MRS/MRI (^1^H: TR/TE = 4.9/2.47 ms, flip angle = 20°, NEX = 1, BW = 50 kHz, FOV = 40×40 mm^2^, ST = 15 mm, coronal, matrix = 128×128; ^19^F (SPGR): TR/TE = 3.46 or 11.71/1.75 or 5.87 ms, flip angle = 50° or 227.5°, NEX = 2048, BW = 10 kHz, FOV = 40×40 mm^2^, ST = 15 mm, coronal, matrix = 32×32).

To facilitate extension of the methodology to the post-mortem mouse, a 3D SPGR acquisition protocol was successfully tested in vitro (flip angle = 200°) using the butterfly coil and reference (800k FuGENE-labeled CPCs) and test (~1.3 million CPCs before their injection—please see (c) below) Eppendorf vials. The two vials were positioned on the surface of the butterfly coil at comparable positions to those used in the post-mortem case. T_1_ and T_2_ correction was achieved based on fully relaxed MRS or the steady-state, closed form, signal equations of SPGR and echo-SSFP, in accordance to Eqs 8 and 16 (S1 Appendix). CPC cell numbers and ^19^F content from samples with unknown content were extrapolated based on known cell densities of reference standards (validated using Trypan Blue), against a 25 mM TFA phantom.

Post-mortem mouse: For these tests, the butterfly coil was used. The reference vial was positioned on the anterior thorax. The cell pellet position was approximately at the same level (along the inferior-superior direction of the animal, inclined ~-45°) as the injection site location. Estimation of the injected cell number was based on the ratio of the total ^19^F MRI signal of the injected cells (upper mid-ventricular myocardium) and the reference CPC signal, obtained using the 3D SPGR adiabatic excitation (optimized in vitro—see (b) above) using a 220° flip angle.

#### Post-mortem and in vivo animal models—Skeletal and cardiac muscle applications

To assess the feasibility and reproducibility of the imaging protocol, labeled cells (~1.5–2.5 million suspended in ~50–100 μl of IMDM media) were injected in the femoral areas of mouse hindlimbs (n = 3), and in the anterior left ventricular (LV) muscle post-mortem (n = 3) (mouse strains included PHD3f/f, PHD2flox/flox, and C57BL/6). The mice were then positioned on the butterfly coil and imaged. ^1^H images were acquired with the 3D SPGR sequence using TR = 2.49 ms, TE = 1.26 ms, flip angle = 20°, NEX = 3, BW = 50–150 kHz, FOV = 40×40 mm^2^, matrix = 192×192×192, in 4.36 min. For ^19^F MRI (3D-SPGR) the acquisition parameters were: TR/TE = 5.64–2.84ms, flip angles = 20°, NEX = 48, BW = 6 kHz, FOV = 40×40 mm^2^, matrix = 32×32×32, in a total of 4.37 min.

#### In vivo cardiac muscle applications

Labeled cells (~1.5 million suspended in ~50 μl of IMDM media) were injected in two C57BL/6J mice (male weight range = 20–30 g) that underwent thoracotomy, followed by recovery. Induction was achieved using 4% ISO, and the mice were maintained with 1.5–2.0% ISO, mixed in 100% oxygen.

The mice were subsequently imaged. All animals were placed on a specially constructed cradle, and were allowed to breathe freely throughout the study. A homeostatically controlled hot-air system was used to maintain mouse body temperature at approximately 37°C. Electrocardiographic (ECG) and breathing rates were monitored using a gating system. Heart rates were maintained at 300–500 beats/min.

Ungated/gated, non-localized ^19^F MRS of the mouse thorax were acquired (following shimming at the ^1^H nucleus) first confirming the presence and resonance of PLGA-NPs (TR = 800–1200 ms, flip angle = 90°, NEX = 32–128, 512 points, BW = 20 kHz). 3D cardiac ^1^H images were then acquired with ungated 3D SPGR (TR/TE = 1–2/2–2.5 ms, flip angle = 10–50°, NEX = 32–128, FOV = 40×40×40 mm^3^, matrix = 128×128×128, BW = 100 kHz) and standard, gated, 2D segmented k-space pulse sequences). Ungated 2D or 3D ^19^F MRI were subsequently acquired (that matched exactly the spatial orientation and FOV of the ^1^H MRI) of the mouse thorax (TR/TE = 9/1 or 16.5/8.3ms, flip angle = 50° or 20–30°, NEX = 768 or 12–32, BW = 2 kHz, FOV = 40×40 mm^2^or 40×40×40 mm^3^, matrix = 128×128 or 32×32×32). The receiver bandwidths of ^19^F MRI ranged between 1.5–2 kHz and allowed spectral selection of the NP vs. ISO peaks. Total ^19^F MRI signals and maximum SNR values were estimated from the focal areas of hyper-enhancement following labeled CPC cardiac injections in mice (two post-mortem and two in vivo).

#### Histology

Post-mortem histological evaluation was performed in mouse hearts on the same day following cellular implantation to confirm CPC localization. In brief, the excised hearts were dehydrated and fixed (in a 4% methanol-free formaldehyde solution), processed, embedded in paraffin, and stored. Serial transverse paraffin sections were subsequently cut, processed, and imaged on a Leica bright-field optical microscope,

#### Image processing

^19^F images were processed in MATLAB (Mathworks, Natick, MA, USA) or ImageJ (NIH, Bethesda, MD, USA), and MRS in CSX (Johns Hopkins, USA) and IDL (Harris Geospatial, USA). The overlay of the ^19^F and ^1^H images was achieved using up-interpolation of ^19^F MRI using bicubic splines followed by merging (at an opacity of 50%) in ImageJ. Flow cytometric data processing and exporting was achieved using FlowJo (FlowJo LLC, Version 10, Ashland, OR, USA).

The field uniformity of the birdcage coil was assessed following high-order shimming (B_0_ homogeneity linewidths of ~30–70 Hz), based on the signal variability (coefficient of variation [CV] = SD/mean) from multiple one-dimensional (1D) profiles spanning the central regions of multiple 2D images (along both image dimensions), acquired using high-concentration TFA phantoms. SNR was estimated as the mean intensity from selected ROIs divided by the background standard deviation.

#### Statistical analyses

All results are reported as mean±standard deviation (SD). Two-tailed Student’s t-tests, were also used (XLSTAT, Addinsoft, New York) to determine whether labeling led to significant changes in transverse relaxation times.

## Results

**Fig 1:**
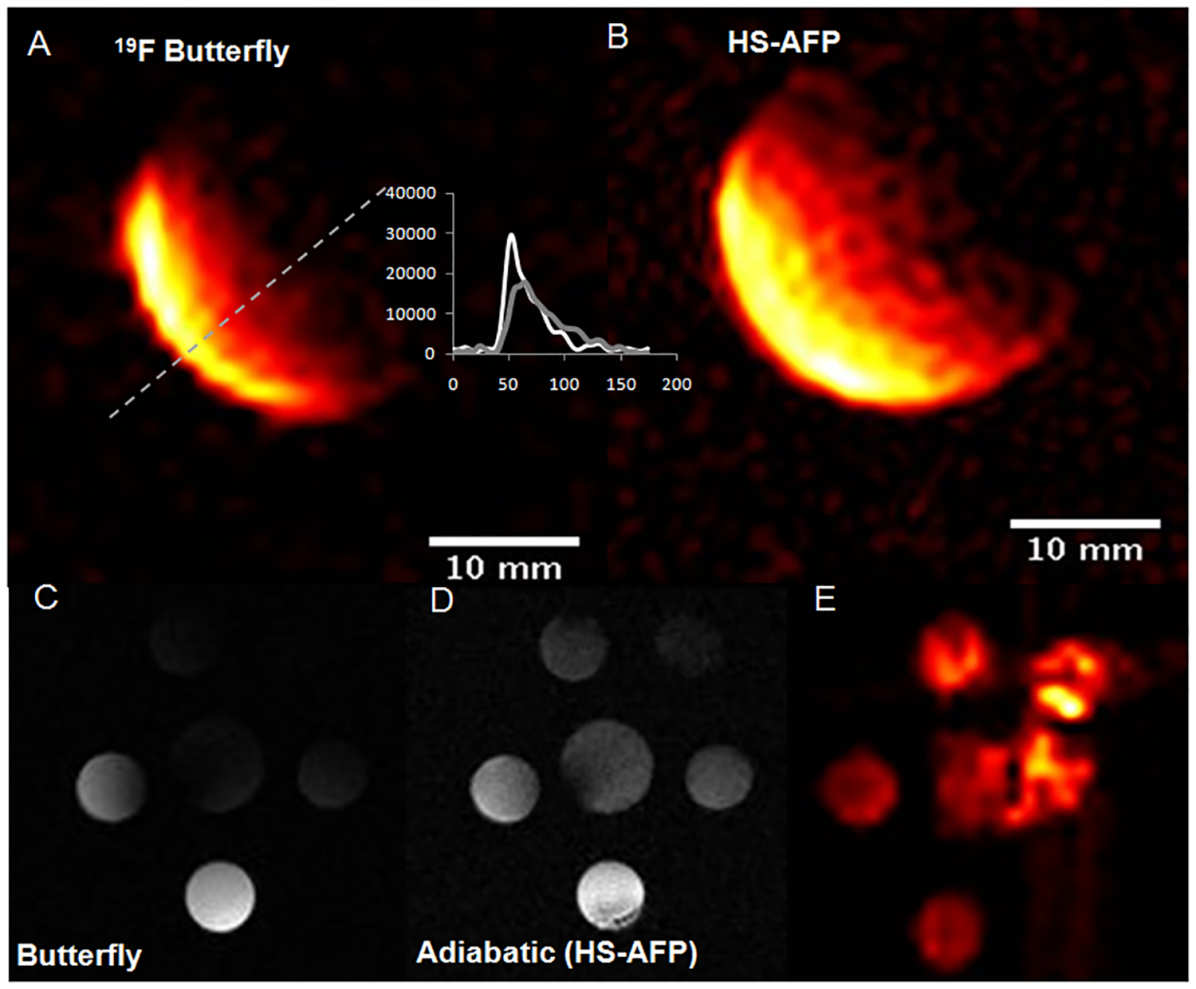
Axial ^19^F MRI images of phantoms using a surface coil. **(A)** butterfly without, and **(B)** with the use of adiabatic hyperbolic secant adiabatic full passage (HS-AFP) using the spoiled gradient echo sequence (SPGR) and a 100 mM TFA phantom. Profiles depict signal intensities versus pixel values along the oblique orientation defined in **A**. **(C-E)** Corresponding axial images of a multivial TFA phantom containing 5 mM TFA solutions. **(C)** Axial ^1^H image using the butterfly coil without, and **(D)** with HS-AFP adiabatic excitation, and **(E)** axial ^19^F with adiabatic excitation. The artifact observed in the right part of the phantom is attributed to fact that the adiabatic condition is not fully met owing to the butterfly’s B_1_ assymetry (driving cables).

Highest field uniformity was achieved by the birdcage coil that yielded a coefficient of B_1_ variation in the central 25×32×32 mm^3^ region of the coil of 2.5% (Fig 1). Additionally, the butterfly yielded the highest SNR, while increased field penetration was noted when adiabatic (HS-AFP) excitation was used. Successful implementation of the adiabatic pulses is also demonstrated with ^1^H and ^19^F MRI of the multivial sensitivity phantom using the butterfly coil, indicating the ability to image ^19^F concentrations of aqueous TFA solutions down to approximately 1 mM, at increased depths of penetration compared to nonadiabatic imaging (Fig 1C–1E).

**Fig 2:**
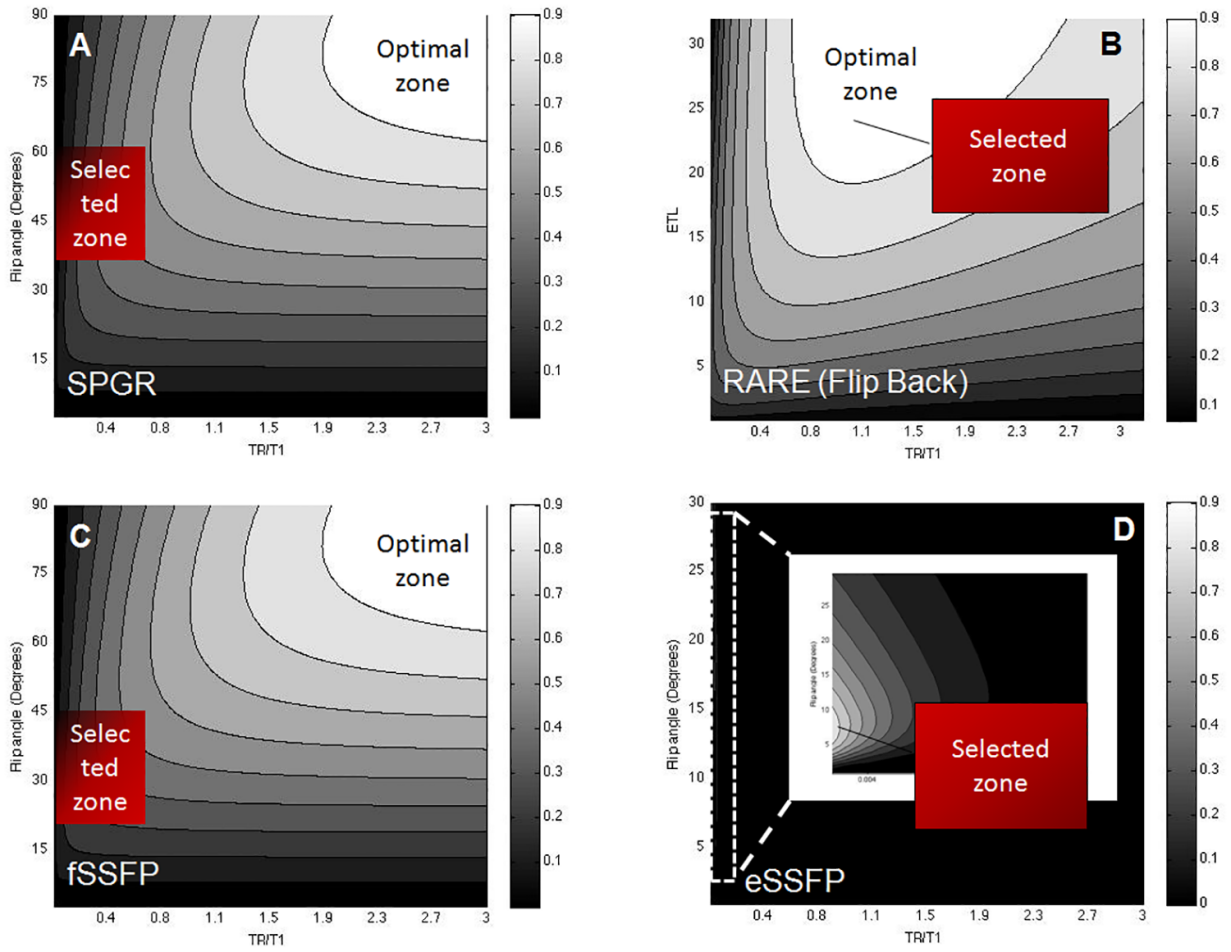
Pulse sequence simulations in parametric space. **(A)** Theoretical normalized parametric signal-to-noise (SNR) plots for labeled CT cells (T_1_ = 1.32 s and T_2_ = 0.05 s) for a: **(A)** SPGR sequence (flip angle versus TR/T_1_), **(B)** a rapid acquisition with relaxation enhancement (RARE) sequence [echo train length (ETL) versus TR/T_1_ with flip-back], **(C, D)** balanced steady state free precession [free induction decay (fSSFP) and echo-SSFP (eSSFP)] (flip angle versus TR/T_1_ without sign alteration). Optimal and selected acquisition zones are indicated. Optimized labeled cell imaging was based on the generation of the respective plots that used the estimated relaxation values as listed in Table 1. All simulations assumed a total imaging acquisition of 4.5 min, NEX = 256, an acquisition matrix of 32×32, and an acquisition bandwidth of 4 kHz.

Normalized parametric SNR maps of the flip angle and ETL versus the normalized TR/T_1_ values for SPGR, RARE, and SSFP imaging, are shown in Fig 2, depicting the zones from which acquisition parameters were selected for fast imaging.

**Fig 3:**
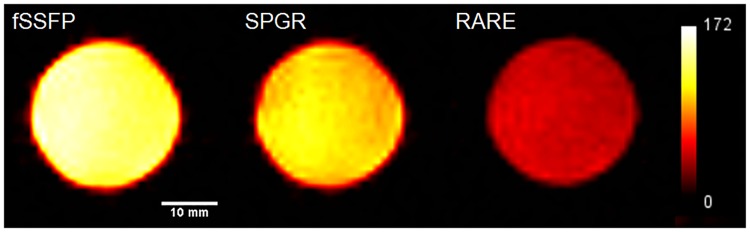
Experimental pulse sequence comparison in phantoms. Pulse sequence SNR comparison using the birdcage coil based on two-dimensional (2D) acquisitions using a 100 mM TFA phantom in the same total imaging acquisition time. SNR values lied within the set scale bar shown on the right.

To allow a direct, experimental comparison of SPGR, RARE, and SSFP sequences, solution phantoms (75–100 mM TFA) were used to perform a direct comparison using the homogeneous birdcage coil (Fig 3). Maximum SNR values (100 mM TFA) are elicited by the fid-SSFP sequence (139±10) that outperformed both the SPGR (106±7) and RARE (48±4) sequences. Based on TFA phantom imaging, SSFP achieved the highest mean SNR (1.3- and 2.9-fold higher than SPGR and RARE) (Fig 3).

T_1_/T_2_ relaxation values for TFA, NaF, NP solutions, and labeled/FuGENE labeled CT cells were 2.73±0.06/2.36±0.16 s, 1.54±0.07/1.11±0.22 s, 0.77±0.02/0.36±0.03 s, 1.32±0.9 s (T_1_), and 1.36 ± 0.09 / 0.53 ± 0.07 s, respectively (Table 1). T_2_ values in labeled cells could not be measured primarily owing to the low ^19^F signal of labeled cells, and the prohibitively long measurement times using CPMG (that would impose cellular viability risks). Labeling led to significant increases in longitudinal relaxation values in CPCs, compared to NPs in media solutions (p<0.00047 (labeled), p<0.001 (FuGENE-labeled), α = 5%).

**Table 1:** In vitro relaxation values of fluorinated compounds. Summary of ^19^F T_1_ and T_2_ relaxation values in phantoms and in labeled cells (immune, neural stem, and cardiac progenitor) from this and prior published studies. Significantly increased T_1_ values (p<0.00047 (labeled), p<0.001 (FuGENE-labeled), α = 5%) were measured for the NP-labeled cells compared to NPs in solution.

Compound	T_1_ (ms)	T_2_ (ms)	Field strength (T)	Comment	Reference
NP (PCE)	1010	526	1.5	PFCE—Gd in lipid monolayer surrounding NP	Neubauer et al. 2008 [32]
NP (Gd)	238	63			
NP (PCE)	990	238	4.7		
NP-Gd	500	24			
NP (PCE)	763.3	71	11.7		
NP-Gd	625	11			
PFCE in dendritic cells	950±30	50±8	7	MRSI—birdcage/solenoid 15 mm in diameter	Bonetto et al. 2012 [33]
Saline	1400±30	440±25	9.4	18 mm surface adiabatic excitation	van Heeswijk 2012 [16]
PCE in venous blood (in vitro)	1350±40	25±2			
Free PFPE	280±20	153±4	11.7	Surface coils 9–25 mm diameter	Boehm-Sturm et al. 2011 [13]
Labeled human neural stem cells	380±4	68±3			
**TFA**	**2728±16**	**2365±18**	**9.4**	**Birdcage/Solenoid**	**This study**
**PLGA-PFCE (solution)**	**773±16**	**360±34**			
**NP labeled CT cells**	**1324±89**	**-**			
**NP FuGENE-labeled CT cells**	**1360±95**	**533±70**			

**Fig 4:**
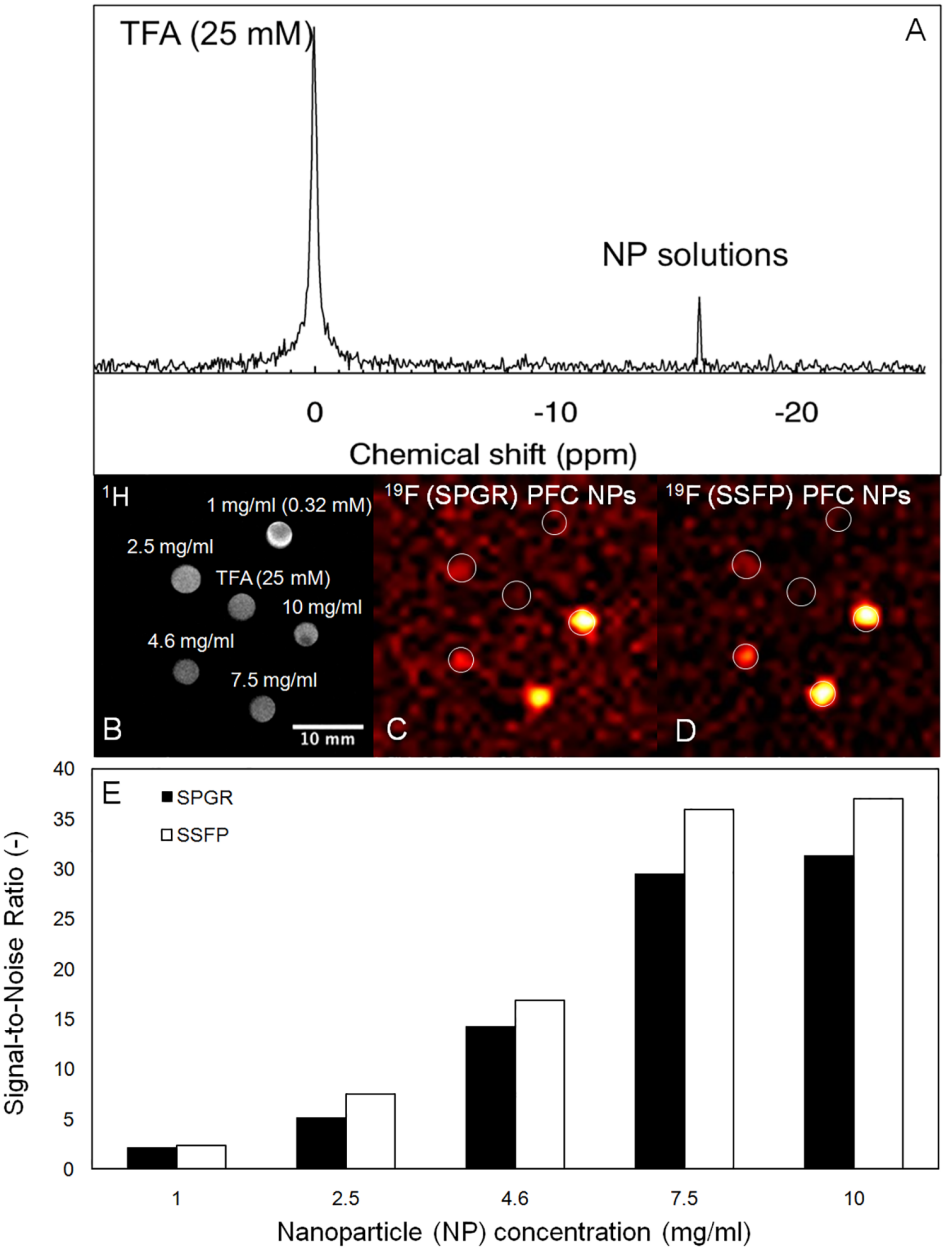
^19^F MRI validation in NP solution phantoms. **(A)** Non-selective ^19^F magnitude spectrum of NP solutions in the presence of a 25 mM TFA phantom (as shown in B). (**B)** ^1^H and ^19^F MRI of NP phantoms using **(C)** the SPGR, and **(D)** the echo-SSFP sequences. The TFA phantom does not appear in ^19^F MRI **(C, D)** since broadband excitation/narrowband receiver detection was used centered at the NP resonance. (**E**) Variation of the mean ^19^F SNR from phantom solutions in **(C, D)** above for the SPGR and SSFP sequences for different NP concentrations.

Based on phantom imaging and MRS, the minimum detectable NP dose was 2.5 mg/ml (0.8mM) (birdcage) (Fig 4). As before, superior SNR performance is demonstrated by echo-SSFP (compared to SPGR) in ^19^F imaging of the NP solutions, whereas lack of substantially improved signals is also documented for NP loading beyond 7.5 mg/ml for the studied CPCs (Fig 4E).

**Fig 5:**
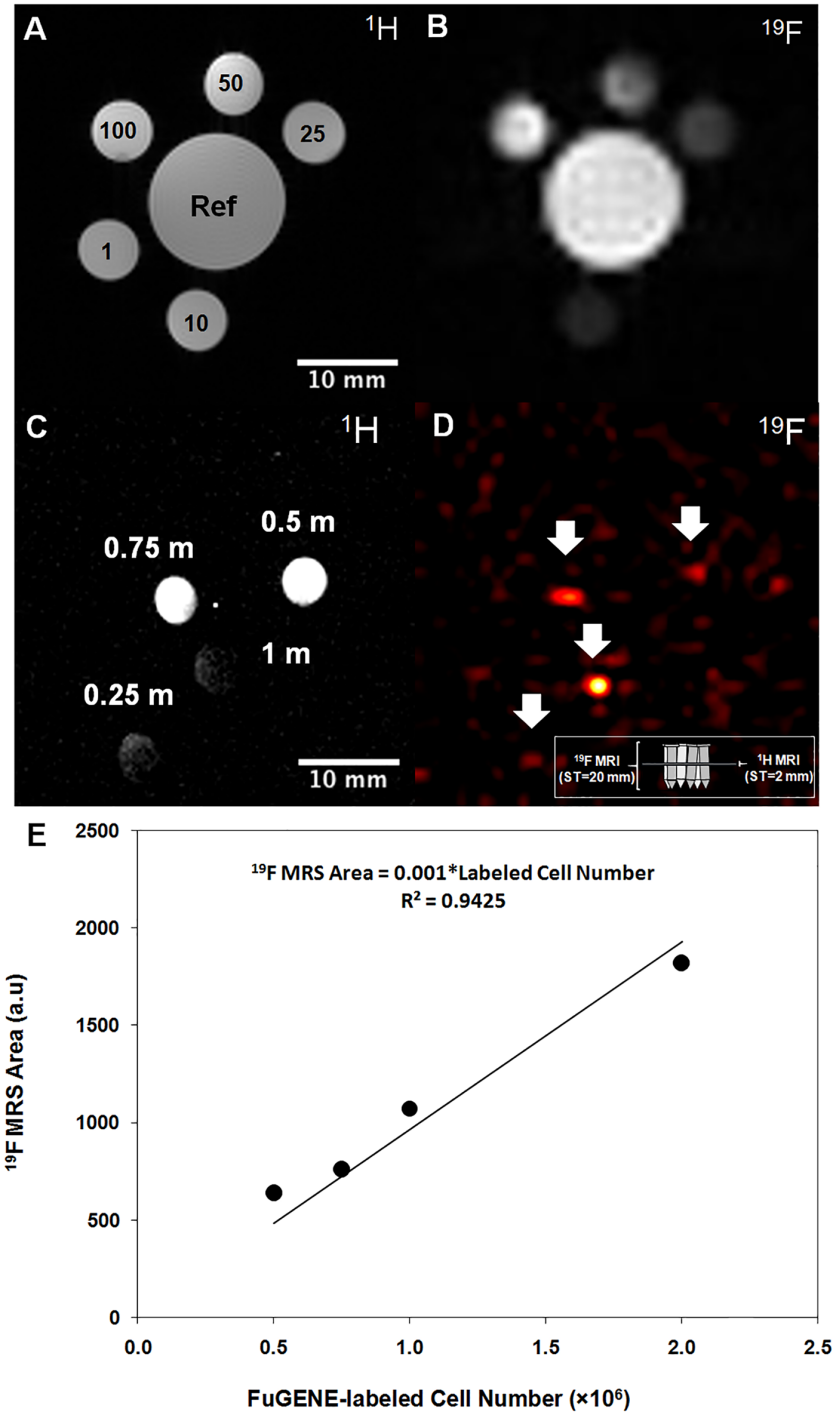
CPC label confirmation using flow cytometry and in vitro ^19^F MRI/MRS validation. **(A, B, D, E)** Ungated scatter plots of forward (FSC) and side scatter (SSC, singlets vs. doublets) and **(C, F)** gated, overlapped flow cytometry histograms of control **(C)** and labeled CT cells **(F)** confirming cellular uptake. Applied gates are indicated in the scatter plots as highlighted regions-of-interest. **(G, H)** Confocal microscopy images of PFCE labelled **(G)** CDC GFP+ (calcein [gray]), **(H)** Atto647 (red), and (**I)** merged calcein/Atto647 with a zoomed inlet indicating the heterogenous distribution of cellular label uptake. **(J, K)** Corresponding ^19^F and ^1^H-^19^F merged MRI of labeled CT cells (~4.5 million) obtained using the solenoid coil showing excellent ^19^F signal localization. **(L)** ^19^F magnitude spectrum in labeled CTs using the solenoid coil (line broadening = 30 Hz, zero reference frequency set to the NP-labeled CT cell resonance).

Fig 5 presents flow cytometry results in justification of the successful labeling protocol. Confocal microscopy images of control and labeled CT cells are shown in (Fig 5A–5F), in justification of the lower sensitivity of detection of the labeled population by confocal microscopy (and correspondingly by MRI). The estimated percentage of viable labeled cells using confocal microscopy was 10% using simple labeling, and significantly increased upon use of FuGENE to 80% or higher [30]. A significant labeling heterogeneity was also noted for CPCs (Fig 5G–5I). Additionally, MRS/MRI allowed imaging and quantification of CPC NP concentrations (fully relaxed MRS for a label loading concentration of 7.5–10 mg/ml/million cells) (Fig 5J–5L) of the order of 0.3–0.5 mM (solenoid).

**Fig 6:**
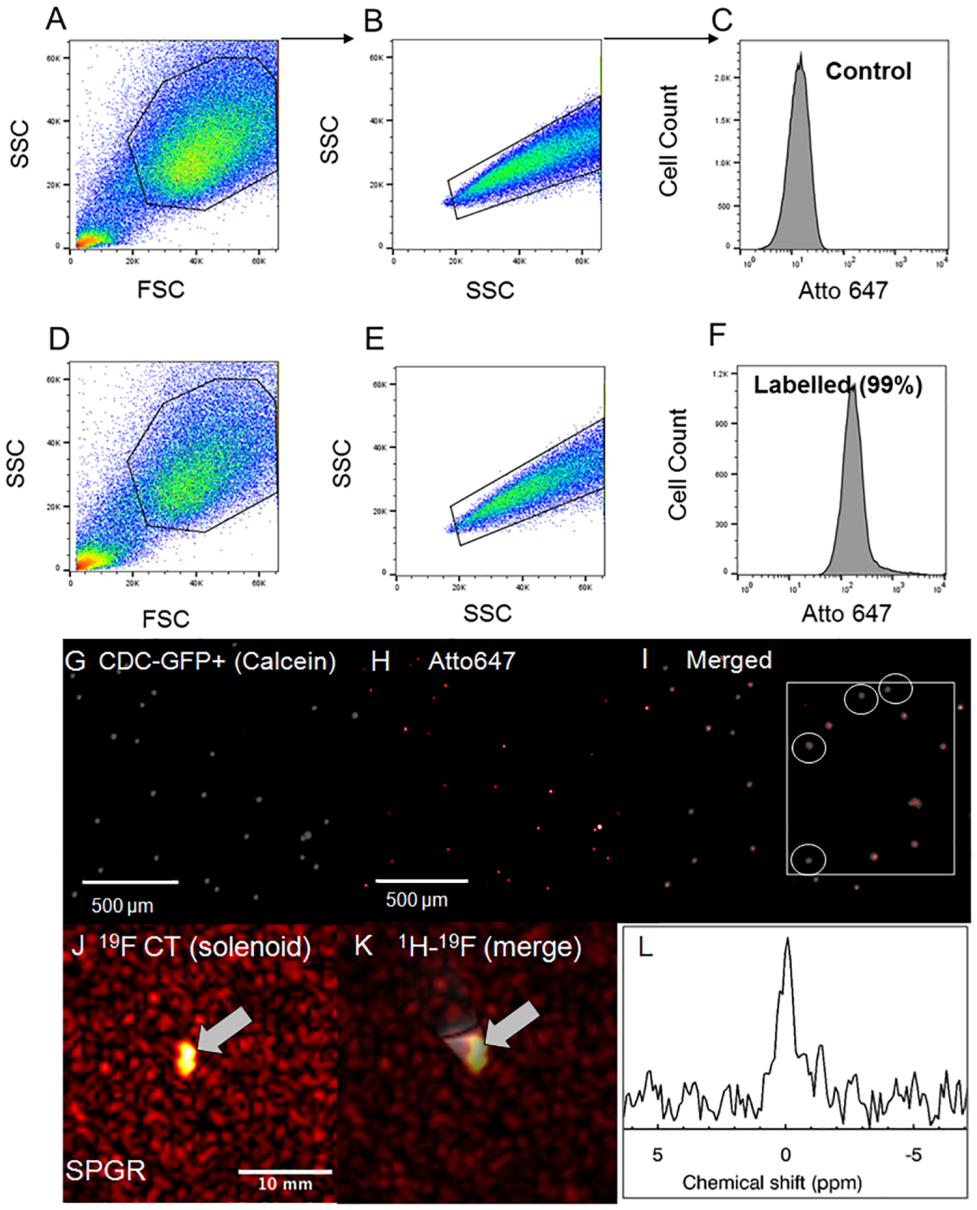
^19^F MRI-based quantification in solutions and CPCs and determination of cellular detectability limit. ^19^F MR spectroscopy, image-based quantification, and sensitivity detection limits: **(A, B)** Axial ^1^H and ^19^F images from TFA phantoms of different concentrations (25–100 mM), and images of a multivial sensitivity phantom containing 0.25, 0.5, 0.75, and 1 million labeled/transfected CT cells suspended in media for sensitivity limit detection (cell pellets resided at the bottom of the Eppendorf tubes) using the butterfly coil. **(C)** ^1^H imaging indicates spatial B_1_ fall off-effects (laterally and with depth, non-adiabatic excitation). ^19^F imaging indicates a minimum detectable cellular load of approximately 500k cells in a total acquisition of 4.4 min (white arrows). The ^19^F MRI in **(D)** shows cells over a slice thickness of 20 mm. As shown by the inserted schematic, the ^1^H MRI in **(C)** shows cross-sections (from the middle of the Eppendorf tubes), while the ^19^F MRI in **(D)** shows the hyperintense cell pellets that were sometimes slightly displaced spatially given the tilting of some of the tubes and the dispersion of the cells on the walls of the tubes in instances where the acquisitions were prolonged. **(E)** Quantification of labelled CPCs using ^19^F MRS (solenoid). The linearity of the evoked fully relaxed spectral area versus cell number was independently confirmed using fast, direct, image-based SPGR using CPCs (butterfly coil) (results not shown).

Image-based quantification (SPGR images, error of ≤ 3%, 10–100 mM) (Fig 6A–6C) confirmed linear signal-concentration dependence in TFA solutions (Actual concentration [mM] = 1.11×Estimated concentration [mM]-5.5, R^2^ = 0.997), with detection limits for the butterfly/birdcage equal to ~0.5 and 10 mM in imaging acquisitions that spanned ~3 min). Fig 6F–6H confirmed that the cellular load detection threshold for the butterfly coil was approximately 0.5 million labeled CPCs (without FuGENE) (Table 2) using the imaging protocol. More importantly, the ability to conduct in vitro, image-based cell quantification was confirmed using TFA phantoms (Fig 6A and 6B), labeled CPC (Fig 6E) and macrophage cells (Actual concentration [mM] = 0.17×Estimated concentration [mM]+0.03, R^2^ = 0.99), using fast imaging and MRS. The effort was extended successfully in the post-mortem mouse using the butterfly coil.

**Table 2:** Comparison of acquisition parameters and cell detection limits in this and prior studies. Optimized sequence parameters for the current ^19^F MRI study led to fast imaging acquisitions (3–5 min) in comparison to prior published studies. The table summarizes the existing literature in reference to the total imaging time, cell detection limit, and the ultimate detection limit for ^19^F signal detection (an extended version of prior published studies can be retrieved from Srinivas et al. [36]).

^19^F label	Cell type	Field strength (T)	Voxe size (μl)	Total imaging (min)	Cell detection limit (per voxel)	^19^F Atom detection limit/voxel (×10^16^)	Reference
PLGA-PFCE	Human dendriti cells	7	2–3.5	27	<30,000	-	Srinivas et al. 2010 [7]
PLGA-PFCE	Human dendritic cells	7	13	60	5000	3.5	Bonetto et al. 2012 [33]
PFCE emulsion	Human SC/progenitor	11.7	27	4	6100	3.8	Partlow et al. 2007 [9]
PFCE emulsion	Murine dendritic cells	9.4	0.1	106	10^6^	170	Waiczies et al. 2011 [34]
PFCE emulsion	Murine macrophages	9.4	0.2–0.4	19	200	0.18	Flogel et al. 2008 [15]
PFCE emulsion	Murine neural SCs	9.4	0.3	5	140 pmol PFCE/cell	-	Ruiz-Cabello et al. 2008 [10]
PCE emulsion	Dendrit c cells	9.4	0.1	70	10^5^	100	Waiczies et al. 2013 [35]
PFC emulsion	Monocytes Macrophages Dendritic cells Granulocytes	9.4	0.4	32	**-**	**-**	Van Heeswijk et al. 2013 [18]
**PLGA-PFCE (no FuGENE)**	**CDC** **CT**	**9.4**	**2**	**3–5**	**10400**	**-**	**This study**

**Fig 7:**
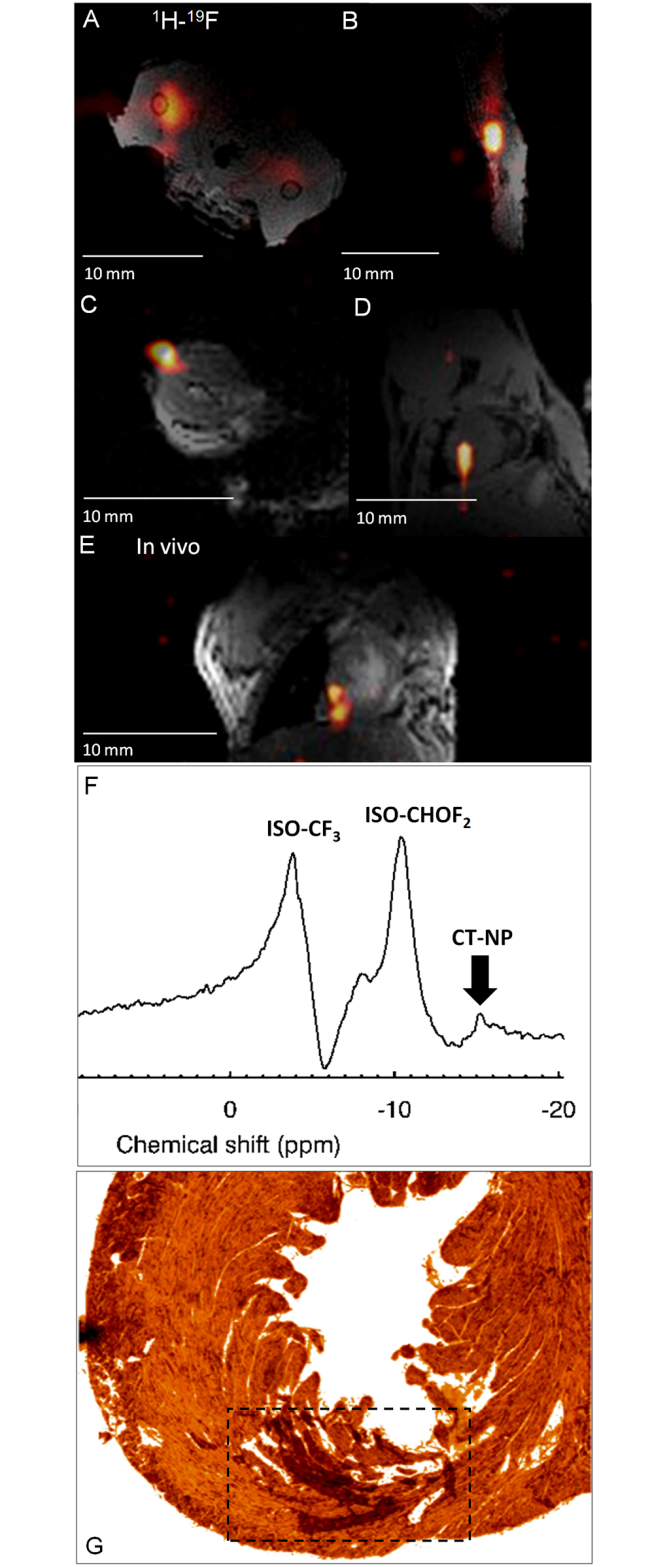
Post-mortem and in vivo murine cardiac ^19^F MRI following intramyocardial CPC injections. Post-mortem **(A-D)** and in vivo **(E-F)**, merged ^1^H-^19^F images of approximately 1.5–2.5 million labeled CT cells administered in the **(A, B)** femoral skeletal (axial, sagittal views; both legs were injected), **(C, D)** post-mortem cardiac (pseudo-short and short-axis views, without **(C)** and with the anterior thorax **(D)**) from PHD3f/f, PHD2flox/flox, **(E)** ungated in vivo cardiac (coronal) views from a C57BL/6 mouse using the butterfly coil. **(F)** Corresponding ungated, unlocalized ^19^F MRS from the upper thorax showing the two isoflurane (ISO) and the labelled CT cell peaks. All ^1^H images were acquired when the coil was tuned/matched at the ^19^F resonance. **(G)** Indicative optical bright field histological image from the mouse heart in **(D)** above. The dotted square box indicates the area where cells were localized within the left ventricular myocardium.

The applicability of the presented methodologies and optimization strategies for in vivo imaging are justified by the fast, post-mortem imaging of injected labeled CPCs in cardiac muscle (in vivo and post-mortem) and femoral areas (post-mortem) of the C57BL/6 mouse [37] (Fig 7). Reported findings were confirmed with histology, whereby cellular injection localization was identified using bright field histological imaging (Fig 7).

From the quantification viewpoint, the in vitro measurement using ^19^F MRI (based on the labeled CPC reference standard) estimated the number of labeled cells as 1.26 million based on the labeled CPC reference standard (compared to the actual number of 1.3 million estimated based on Trypan Blue). Only 1 million cells were quantified from the post-mortem images. The discrepancy is attributed to injection losses (resuspension fluid loss before injection, and other injection losses).

From the post-mortem and in vivo imaging viewpoints, the total ^19^F signal of injected CPCs in mice (post-mortem vs. in vivo) yielded a CV of 24%, whereas the maximum SNR values were comparable (10±1.8, range = 8–11.8). The reported variability is a result of the expected discrepancies in the injected number of cells and injection losses in the four studied cases.

## Discussion

Despite the technical, biological, and imaging advances and development of ^19^F MRI [5, 6, 9, 10, 15, 33, 38] and cellular tracking during the last decade [39, 12, 13, 24, 35, 40], no prior imaging and quantification work focused on CPCs [12, 13, 36].

While Faber and Schmid [26] have recently reported theoretical and experimental comparisons of ^19^F pulse imaging strategies, the approach adopted herein is mathematically rigorous and analytic, and includes reference to the explicit mathematical formulations, allowing theoretical comparison of SSFP, SPGR, and RARE sequences. Additionally, the 2D/3D comparison is also formulated mathematically, thereby justifying the efficiency and expected SNR improvements in 3D (as tabulated in Faber and Schmid [26]). Additionally, there are important practical implications relevant to the translatability and implementation of the bSSFP sequence for cardiac ^19^F MRI that have not been addressed previously as they pertain to artifacts and SNR performance for exogenously administered SCs in the in vivo murine beating heart, while the explicit mathematical formulation for the signal of the SSFP sequence (fid vs. echo) is lacking (useful and relevant for quantification). Furthermore, there have been no prior direct, experimental comparisons of image SNR under controlled phantom conditions for SPGR, SSFP, and RARE. Prior publications on the performance of SPGR imaging in ^19^F MRI, including the recent work by Faber and Schmid [26], have shown merit only for Ernst angle imaging. Nevertheless, in this work, the inefficient aspects of (slow) Ernst angle and the beneficial aspects of fast SC imaging are emphasized using SPGR.

Technical development of cardiac ^19^F MRI using exogenously administered SCs has been lacking, while optimizations of MR imaging acquisitions in association with SCs have been limited [26].

More importantly, imaging times for adopted methodologies in most applications in vivo were excessively long (often > 60 min) [36] due to the low cellular label concentration and the choice of the imaging sequences, often becoming prohibitive for translational work. Interestingly, recent implementations of compressed sensing in ^19^F MRI [41, 42] have led to reduced imaging times, albeit limited by the low SNR of multinuclear in vivo studies.

We have presented the first in vivo cardiac ^19^F MRI data from labeled CPCs injected in the murine myocardium. We have demonstrated feasibility and reproducibility of fast (of the order of a few minutes), in vivo cardiac ^19^F-MRI of exogenously administered CPCs in the murine heart using SPGR/SSFP sequences.

The imaging protocol can be easily and readily adopted for any other labeling agent in preclinical work, and has potential for use in translational work. Additionally, the presented theoretical and experimental schemes are label-independent and can be readily applied to any other fluorinated compounds.

In contrast to imaging of nuclei other-than-protons (e.g., ^23^Na MRI), where the intrinsically abundant sodium nucleus exhibits fast longitudinal relaxation (and where the optimal SNR is elicited at the Ernst angle favoring TR≈T_1_), ^19^F MRI depends on exogenously administered NPs, emulsions, or labeled cells, with relatively long T_1_ values and small T_2_/T_1_ ratios. Despite the expected maximal SNR increases for SPGR sequences at long TRs, it is recommended that fast acquisitions are used in conjunction with averaging and with an appropriate choice of the flip angle, based on theoretical evaluations. This study has also assessed the cellular load detection thresholds (0.5 mM for the butterfly versus 10 mM for the birdcage coil) for ^19^F NP labels, based on fluorinated phantom solution comparisons and fast acquisitions of the order of a few minutes at voxel resolutions of ~2 μl or less.

Despite recent efforts to chemically modify the T_1_/T_2_ characteristics of labeled NPs [43] to speed up acquisitions, such approaches require complex chemical syntheses. The PLGA-PFC NPs used in this study have elicited T_1_ and T_2_ values that are in agreement with prior reports [36], and a T_1_-effect is demonstrated post-cellular loading. The inability to quantify T_2_ upon cellular loading is primarily attributed to the low labeling efficiency (and hence the low ^19^F signal), and to the intracellular endosomal/vesicular packaging of the NP label leading to a relatively short transverse relaxation times (of the order of a few ms) [39, 36]. Correspondingly, prolonged spectroscopic acquisition times will invariably impose additional issues in terms of the cellular oxygenation status (oxygen tension, leading to hypoxia [41]), and altered viability, compromising T_2_ estimates. Given the limited in vivo/post-mortem visibility of labeled cells (without FuGENE), and the reported dependence of the T_2_ values of PFCE-NPs on i) temperature [44, 45], ii) label concentration [46], and iii) cell type, and the iv) extremely low ^19^F signal of labeled cells, increased complexity and limited usefulness and consistency, are anticipated from the measurement of these values.

Our reported relaxation values in NP solutions (T_1_ and T_2_) are smaller in value than those recently reported by Colotti et al. [46] at 24°C at 3T. This difference, however, is expected in view of the B_0_-field relaxation dependence trends reported by de Vries et al. for ^19^F emulsions [44].

Our successful labeling protocol was confirmed using flow cytometric and confocal microscopy validations. Based on validation studies presented herein, no additional benefits are expected by increasing the label concentration beyond 7.5–10 mg/ml per million cells, given the constancy of elicited signal responses in NP solutions using both the SPGR and SSFP acquisitions.

The minimum cellular detectable load (no FuGENE) was determined to be approximately 500k cardiac stem cells (or equivalently~10k cells per voxel) in fast acquisitions (~3–5 min) using the butterfly coil. This finding can be justified by the inefficient process of cellular label uptake in these cardiac stem cells in association with their much smaller cellular size (~30 μm^3^ isotropic) compared to dendritic cells or macrophages. Evidently, FuGENE significantly decreases this detection limit, thereby achieving ^19^F cardiac MRI [30].

Regarding quantification, we have demonstrated direct spectroscopy, and direct image-based quantification of absolute ^19^F concentration in TFA solutions, in labeled CPCs and macrophage cells, and injected cells post-mortem, with responses that scale linearly with increased fluorinated label or fluorine concentrations ex vivo. The ability to conduct both ^19^F MRI/MRS provides an easy/efficient methodological pathway to study focal myocardial disease.

However, unlike prior quantification studies [6, 47, 48], a major limitation of the quantitative capacity of in vivo ^19^F MRI is the fact that it cannot be applied to CPCs given their low and heterogeneous label uptake at insufficient levels for their MRI detection. To overcome such a limitation, we have validated an in vitro quantification scheme (in combination with adiabatic excitation) that can be adopted to in vivo applications, where a secondary reference phantom containing a known number of labeled CPCs can be used.

The present study is associated with various limitations, including the focus on two particular CPC types that consist of a heterogeneous cell mixture, and their low label uptake that ultimately hinders T_2_ measurements. Given the stringent spatial requirements of the high-field bore system, temperature and oxygen tension effects cannot be easily monitored during relaxation measurements, or following cellular injection. Furthermore, our study has focused on PFCE with a single resonant peak, compared to other fluorinated compounds (e.g., PFOB) that exhibit complex, multi-peak responses.

Another major limitation is the lack of quantitative accuracy in vivo primarily owing to the stringent time limitations for additional data acquisitions for achieving adiabaticity and B_1_ and motion corrections. Even still, an additional confounding factor that may limit quantification is the diminished viability of injected cells as a result of the hypoxic environment in which they are injected [1]. Correspondingly, evoked ^19^F signal hyper-enhancements that may be attributed to viable cells, or released NPs from lysed cells (immediately following injection, or in tracking studies), ought to be interpreted with care. Such present the primary limitations in translating this work to a true experimental setting in humans.

Furthermore, the spatio-temporal effects of motion on ^19^F cardiac MRI may need to be addressed in more detail. However, preliminary tests with the implemented protocols indicate lack of spatial discrepancies between ungated and gated ^1^H MRI scans (voxel size = 0.2 μl) in normal mice. Correspondingly, possible motion effects between ungated and gated ^19^F MRI acquisitions are expected to be minimal, considering the large voxel (voxel volume>2 μl), and the spatio-temporal averaging over the acquisition time intervals. Although gated cardiac ^19^F scans are possible, they are prohibitively long (exceeding at least 30 min, thereby imposing beat-to-beat variability issues) and yield inadequate image SNR responses that would disallow NP detectability and localization.

## Supporting information

S1 Appendix Theoretical background: MRI signal-to-noise ratio maximization: Theoretical and experimental considerations. (DOC)
