# Correction: Fast, quantitative, murine cardiac ^19^F MRI/MRS of PFCE-labeled progenitor stem cells and macrophages at 9.4T

**DOI:** 10.1371/journal.pone.0225786

**Published:** 2019-11-21

**Authors:** Christakis Constantinides, Mahon Maguire, Eileen McNeill, Ricardo Carnicer, Edyta Swider, Mangala Srinivas, Carolyn A. Carr, Jurgen E. Schneider

The images for Figs 5 and 6 are incorrectly switched. The image that appears as Fig 5 should be Fig 6, and the image that appears as Fig 6 should be Fig 5. The figure captions appear in the correct order.

**Fig 5:**
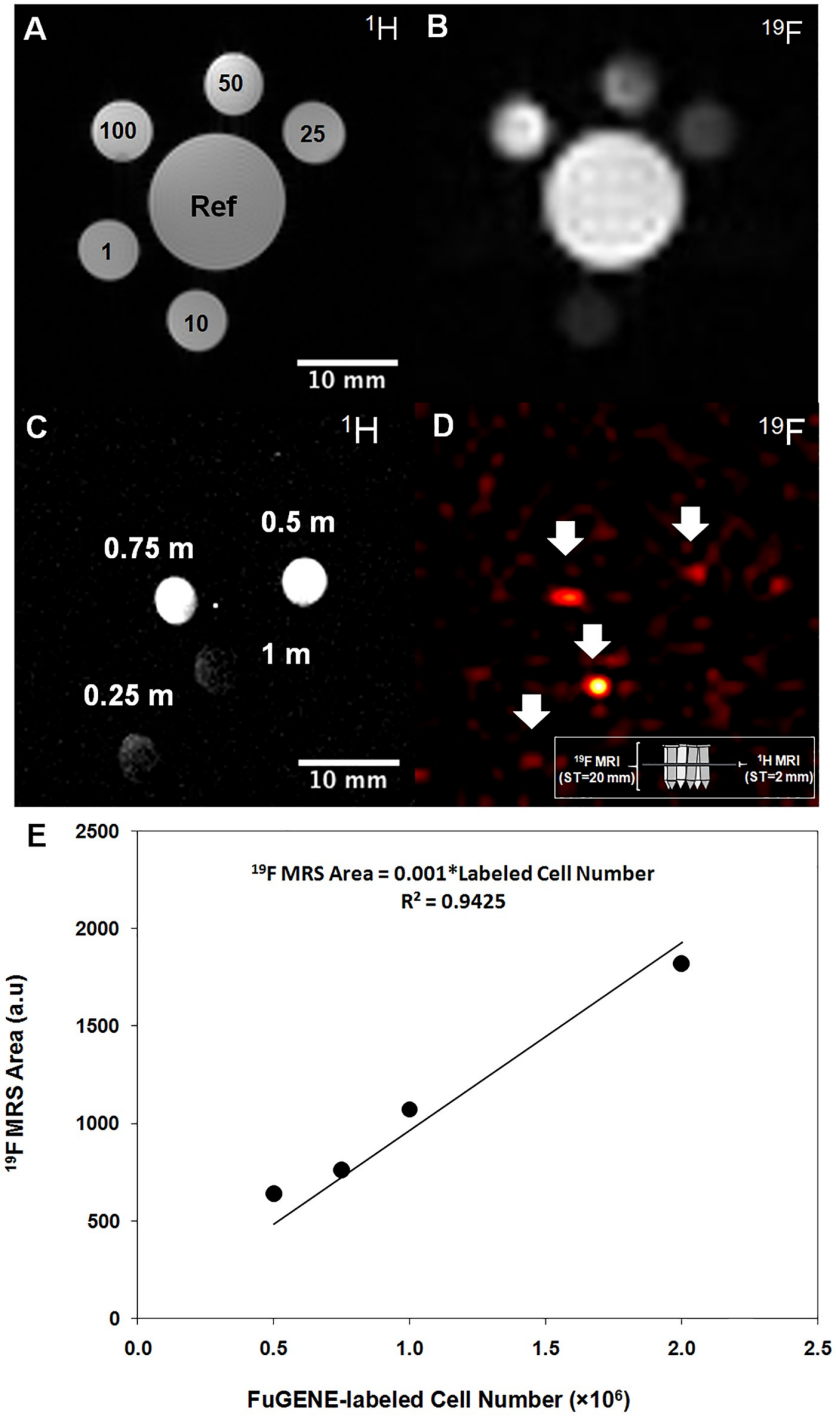
CPC label confirmation using flow cytometry and in vitro ^19^F MRI/MRS validation. **(A, B, D, E)** Ungated scatter plots of forward (FSC) and side scatter (SSC, singlets vs. doublets) and **(C, F)** gated, overlapped flow cytometry histograms of control **(C)** and labeled CT cells **(F)** confirming cellular uptake. Applied gates are indicated in the scatter plots as highlighted regions-of-interest. **(G, H)** Confocal microscopy images of PFCE labelled **(G)** CDC GFP+ (calcein [gray]), **(H)** Atto647 (red), and **(I)** merged calcein/Atto647 with a zoomed inlet indicating the heterogenous distribution of cellular label uptake. **(J, K)** Corresponding ^19^F and ^1^H-^19^F merged MRI of labeled CT cells (~4.5 million) obtained using the solenoid coil showing excellent ^19^F signal localization. **(L)** ^19^F magnitude spectrum in labeled CTs using the solenoid coil (line broadening = 30 Hz, zero reference frequency set to the NP-labeled CT cell resonance).

**Fig 6:**
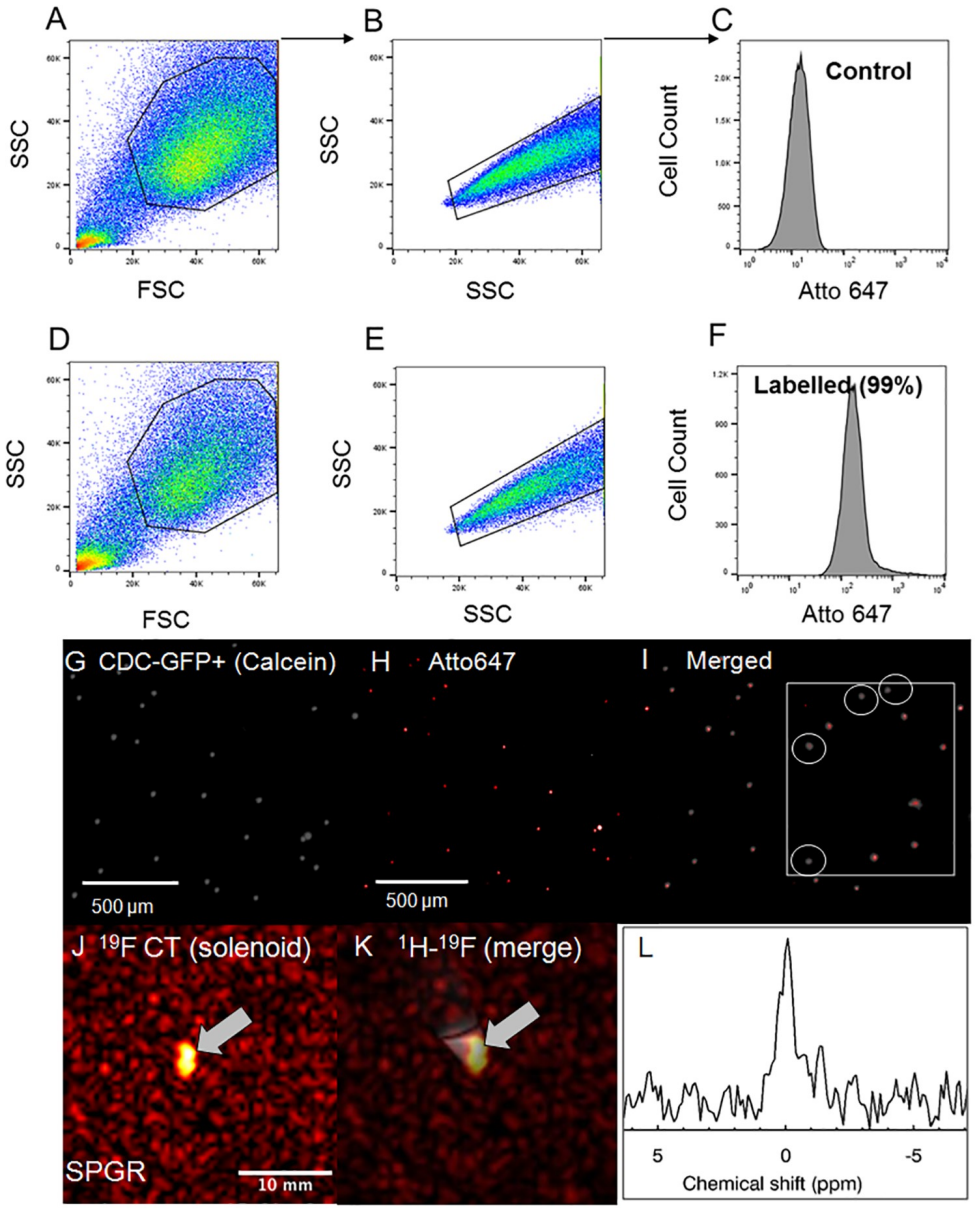
^19^F MRI-based quantification in solutions and CPCs and determination of cellular detectability limit. ^19^F MR spectroscopy, image-based quantification, and sensitivity detection limits: **(A, B)** Axial ^1^H and ^19^F images from TFA phantoms of different concentrations (25–100 mM), and images of a multivial sensitivity phantom containing 0.25, 0.5, 0.75, and 1 million labeled/transfected CT cells suspended in media for sensitivity limit detection (cell pellets resided at the bottom of the Eppendorf tubes) using the butterfly coil. **(C)** ^1^H imaging indicates spatial B_1_ fall off-effects (laterally and with depth, non-adiabatic excitation). ^19^F imaging indicates a minimum detectable cellular load of approximately 500k cells in a total acquisition of 4.4 min (white arrows). The ^19^F MRI in **(D)** shows cells over a slice thickness of 20 mm. As shown by the inserted schematic, the ^1^H MRI in **(C)** shows cross-sections (from the middle of the Eppendorf tubes), while the ^19^F MRI in **(D)** shows the hyperintense cell pellets that were sometimes slightly displaced spatially given the tilting of some of the tubes and the dispersion of the cells on the walls of the tubes in instances where the acquisitions were prolonged. **(E)** Quantification of labelled CPCs using ^19^F MRS (solenoid). The linearity of the evoked fully relaxed spectral area versus cell number was independently confirmed using fast, direct, image-based SPGR using CPCs (butterfly coil) (results not shown).

S1 Appendix erroneously contains annotated comments. Please view the correct S1 Appendix below.

## Supporting information

S1 Appendix Theoretical background: MRI signal-to-noise ratio maximization: Theoretical and experimental considerations. (DOC)
