# Supplementary material for: Fast, quantitative, murine cardiac 19F MRI/MRS of PFCE-labeled progenitor stem cells and macrophages at 9.4T
Source: PLoS One. 2018 Jan 11;13(1):e0190558. doi: 10.1371/journal.pone.0190558 (PMC5764257; doi:10.1371/journal.pone.0190558)
Supplement: S1 Appendix — (DOC) [file pone.0190558.s001.doc]

**Supporting Information**

**S1 Appendix**

**Theoretical background: MRI signal-to-noise ratio maximization: theoretical and experimental considerations**

**Pulse Sequences**

Analytical, closed-form SNR expressions for various sequences (SPGR, RARE, fid-SSFP, echo-SSFP) are summarized herein. Preliminary tests and validations are confined to 2D, but can be readily extended to 3D, as indicated herein.

In accordance to basic MRI principles, SNR can be formulated as

(1)

$$SNT\propto\Delta V\sqrt{T_{acq,total}}S(T_{1},T_{2},TR,TE,\theta)$$

where ΔV is the sample volume, and T_acq,total_ is the total data acquisition time considered as the time during which the data acquisition window is open during each readout (equal to the number of frequency encoding steps, N_freq_, divided by the receiver bandwidth (BW)) [1],

$$T_{acq,total}=N_{freq}.T_{sampling}=\frac{N_{freq}}{BW}$$

(2)

T_1_ and T_2_ the respective relaxation values, and θ is the flip angle. $S(T_{1},T_{2},TR,TE, \theta)$is a composite function that determines the dependence of the evoked signal on relaxation times and pulse sequence acquisition parameters. Assuming constancy of B_1_, and adaptation of the formulation for a generic 2D/3D pulse sequence yields:

$$SNR_{2D}\propto\Delta V.S(T_{1},T_{2},TR,TE,\theta).\sqrt{N_{ph1}.NEX.T_{acq,total}}$$

(3])

(4)

where N_ph1_ and N_ph2_ are the phase encoding steps along the second and third encoding directions, respectively. Correspondingly, Eqs 3 and 4 transform to

(5)

(6])

Combination of Eqs 5 and 6 results in

$$\frac{SNR_{3D}}{SNR_{2D}}=\sqrt{N_{ph2}}$$

(7)

**2D and 3D Gradient Echo**

The steady-state, closed-form expressions for a conventional SPGR sequence (when B_1_ effects are ignored) is given by

$$SNR_{SPGR}(T_{1},T_{2},TR,TE,\theta)\propto\frac{M_{o}\left(1-e^{-TR/T_{1}}\right).e^{-TE/T_{2}^{*}}\sin\theta}{1-\cos\theta.e^{-TR/T_{1}}}$$

(8)

where $T_{2}^{*}$ is introduced to account for the increased susceptibility of gradient-echo sequences to B_0_ inhomogeneities. Typically, interleaved multi-slice acquisitions are used for 2D imaging (with single-slice 2D representing the trivial simplification of such acquisitions). If the differences in the Ernst angles of 2D and 3D acquisitions and the increase in TR due to the N interleave, multi-slice acquisition scheme, are considered, it can be shown that [1],

$$\frac{SNR_{3D}}{SNR_{2D}}=\sqrt{N_{ph2}}.\frac{\sqrt{1-e^{-2NT_{acq,total}/T_{1}}}}{\sqrt{1-e^{-2T_{acq,total}/T_{1}}}}.\frac{\left(1-e^{-T_{acq,total}/T_{1}}\right)}{\left(1-e^{-NT_{acq,total}/T_{1}}\right)}$$

(9)

where it is assumed that the 2D and 3D acquisitions use the same TE, and that the $T_{2}^{*}$ dependence can thus be ignored.

In consideration of Eq 9, and in the effort to maximize SNR, there is a practical limit to which the BW can be decreased owing to susceptibility effects. This effect worsens in vivo and with the administration of labeled compounds.

Furthermore, consideration of Eq 9 indicates that it is important to distinguish differences in the SNR ratio in fast (T_acq,total_<<T_1_) and slow (T_acq,total_>>T_1_) scans, given the number of acquired slices and the value of T_acq,total_/T_1_. Based on prior work [1, 2], the SNR of 2D and 3D acquisitions are comparable at different number of slices and T_acq,total_/T_1_values (especially for Ernst angle excitations and for 2D acquisitions where TR<T_1_). Three-dimensional acquisitions become more favored for increased numbers of slices owing to their efficient spatial coverage, however, this may be irrelevant in ^19^F MR imaging and cellular tracking owing to the limited amount of cells and physiological complexities associated with the fate of the injected cells in vivo. However, SNR improvements of the order of are expected in 3D acquisitions (compared to 2D) in slow, quantitative (fully relaxed) scans (T_acq,total_>>T_1_).

**2D and 3D RARE**

RARE [3] exhibits a number of advantages compared to other imaging acquisition techniques, and in ^19^F MRI the flip-back and no-flip back cases have been distinguished [4, 5]. In accordance to this work,

(10)

(11)

where ΔΤΕ is the echo spacing, ETL is the echo train length (the number of echoes acquired during each repetition), S_ave_ is the average signal at steady state, is the value of the steady state magnetization at the beginning of the pulse sequence repetition, and n is the number of refocusing pulses. Correspondingly, the closed-form SNR equations in 2D and 3D are given by

$$SNR_{2D,RARE}\propto\Delta V.\frac{1}{ETL}\sum_{n=1}^{ETL} M_{z}^{0}\left(1-e^{-\left[\left(TR-(ETL.\Delta TE)/T_{1}\right)\right]}\right).e^{-n\Delta TE/T_{2}}.\frac{\sqrt{N_{ph1}.N_{freq}.NEX}}{\sqrt{BW}}$$

(12)

(13)

where N_ph2_ is the number of additional phase encoding steps (along the third encoding direction). Thus,

$$\frac{SNR_{3D}}{SNR_{2D}}\propto\sqrt{N_{ph2}}$$

(14)

indicating that there is a clear advantage of 3D RARE compared to 2D RARE.

**2D and 3D SSFP**

Herein, two classical variants of the SSFP are considered, namely: a) fid-, and b) echo-SSFP that can execute with sign alteration, and without [6]. Correspondingly, the closed form signal expressions for fid and echo-SSFP are [7]

$$Signal_{SSFP-Fid}=M_{o}.\tan\left(\frac{\theta}{2}\right)\left[1-\frac{\left(e^{-TR/T_{1}}\text{ }-\cos\theta\right).\left(1-e^{-2TR/T_{2}}\right)}{\sqrt{p^{2}-q^{2}}}\right]$$

(15)

(16)

where$p=1-e^{-TR/T_{1}}.\cos\theta-e^{-2TR/T_{1}}\left(e^{-TR/T_{1}}-\cos\theta\right)$ and $q=e^{-TR/T_{2}}.(1-e^{-TR/T_{1}}).(1+\cos\theta)$.

Additionally, use of the Ernst condition for short TR SSFP acquisitions with sign-alternation (TR<<T_1_, as suggested herein for ^19^F MRI) yields [7]:

(17)

Similarly, the ratio of Eqs 16 and 15 for short TR acquisitions leads to [1]

$$\frac{Signal_{Echo}(t)}{Signal_{Fid}(t)}\approx e^{-2TR/T_{2}}$$

(18)

Therefore, considering prior published ^19^F relaxation values in labeled cells [8], the reported results of this study (Table 1), and Eqs 17–18, it can be implied that SSFP can yield signals that are less than 15–36% of the total magnetization, while fid- and echo-SSFP sequences elicit signals with similar maximum values.

Extension of the theoretical analysis in 3D follows similar arguments as those presented for SPGR and RARE above, and yields

$$SNR_{2D,Fid-SSFP}\propto\Delta V.\frac{\sqrt{N_{ph1}.N_{freq}.NEX}}{\sqrt{BW}}.M_{o}.\tan\left(\frac{\theta}{2}\right)\left[1-\frac{\left(e^{-TR/T_{1}}\text{ }-\cos\theta\right).\left(1-e^{-2TR/T_{2}}\right)}{\sqrt{p^{2}-q^{2}}}\right]$$

(19)

(20)

Equivalent arguments can be applied for the formulation of the 3D Echo-SSFP equation and the ratio of SNR_3D,Fid-SSFP_/SNR_2D,Fid-SSFP_.

**References**

1. Handbook of MRI pulse sequences. Bernstein, King, and Zhou. Handbook of MRI pulse sequences. London: Elsevier 2004. 424–431, 583–591, 777–795 p., 2004.

2. Johnson H, Wadghiri YZ, Turnbull DH. 2D multislice and 3D MRI sequences are often equally sensitive. Magnetic Resonance in Medicine 1999; 41:824–828.

3. Hennig J, Nauerth A, Friedburg H. RARE imaging: a fast imaging method for clinical MR. Magnetic Resonance in Medicine 1986; 3:823–833.

4. Mastropietro A, De Bernadi E, Breschi GL, Zucca I, Cametti M, Soffientini CD, et al. Optimization of rapid acquisition with relaxation enhancement (RARE) pulse sequence parameters for ^19^F MRI studies. Journal of Magnetic Resonance Imaging 2014; 40:162–170.

5. Faber C, Schmid F. Pulse sequence considerations and schemes. In: Flogel U, Ahrens ET, editors. Fluorine Magnetic Resonance Imaging. Singapore: Pan Stanford Publishing: 2016; 1–28.

6. Duerk JI, Lewin JS, Wendt M, Petersilge C. Remember true FISP? A high SNR, near 1-second imaging method for T2-like contrast in interventional MRI at .2T. Journal Magnetic Resonance Imaging 1988; 8:203–208.

7. Hanicke W, Vogel HU. An analytical solution for the SSFP signal in MRI. Magnetic Resonance in Medicine 2003; 49:771–775.

8. Srinivas M, Heerschap A, Ahrens ET, Figdor CG, de Vries IJM. ^19^F MRI for quantitative in vivo cell tracking. Trends in Biotechnology 2010; 28(7):363–370.
